# Diagnosis and Surgical Repair for Coarctation of the Aorta With Intracardiac Defects: A Single Center Experience Based on 93 Infants

**DOI:** 10.3389/fped.2020.00049

**Published:** 2020-03-03

**Authors:** Yuhao Wu, Jiashan Li, Chun Wu, Jin Zhu, Ling He, Chuan Feng, Yiting Yang, Xin Jin

**Affiliations:** ^1^Department of Cardiothoracic Surgery, Children's Hospital of Chongqing Medical University, Ministry of Education Key Laboratory of Child Development and Disorders, China International Science and Technology Cooperation Base of Child Development and Critical Disorders, National Clinical Research Center for Child Health and Disorders, Chongqing Key Laboratory of Pediatrics, Chongqing, China; ^2^Department of Pathology, Chongqing Medical University, Chongqing, China; ^3^Department of Radiology, Children's Hospital of Chongqing Medical University, Chongqing, China

**Keywords:** congenital heart disease, coarctation of the aorta, surgery, infants, outcomes

## Abstract

**Introduction:** This study aims to summarize the clinical characteristics of coarctation of the aorta (CoA) associated with intracardiac anomalies in infants.

**Methods:** Medical records of 93 infants who were diagnosed with CoA and intracardiac anomalies from August 2009 to August 2018 were retrospectively reviewed.

**Results:** All of the 93 infants underwent single-stage repair for CoA associated with intracardiac anomalies. The mean operative time was 264.6 ± 57.1 min, and the time of ICU stay was 7.0 ± 4.1 days. The residual transcoarctation pressure gradient before discharge was lower than the pressure gradient prior to surgery (48.3 ± 17.5 vs. 22.4 ± 9.6 mmHg, *P* < 0.01). Early death before discharge was found in five infants. The mean follow-up time of 88 hospital survivors was 40.0 ± 26.4 months, and no subsequent death occurred in the follow-up. Transcoarctation pressure gradient of the 88 survivors in their last follow-up was 19.6 ± 10.5 mmHg. The pressure gradient of 27 cases was higher than 20 mmHg. Significant lower limb retardation was observed in four cases; therefore, balloon angioplasty consult was recommended. The cumulative recoarctation-free survival rate in 3-year follow-up was 73.5%.

**Conclusions:** To avoid secondary operations in early period, single-stage repair of CoA associated with intracardiac anomalies was effective and safe, and the outcomes in early to mid-term follow-up were satisfactory.

## Introduction

Coarctation of the aorta (CoA) is an unusual congenital heart defect, which accounts for 5–8% in all congenital heart diseases (1–3). The most commonly associated intracardiac defect of CoA is the ventricular septal defect (VSD) (4, 5). Poor outcomes are often observed in patients with CoA and intracardiac defects in early age; therefore, surgery should be performed in infants or even in neonates. A recent study (6) indicated that no significant difference was found in early mortality and the incidence of recoarctation between single-stage and two-stage repair_._ Therefore, most medical centers currently prefer single-stage repair for CoA with intracardiac defects (7). This study aims to review the data regarding single-stage repair of CoA and intracardiac defects in our institution and provide unambiguous evidence that approves the efficacy and safety of single-stage repair.

## Materials and Methods

### Patients Demographics and Definitions

A review of the medical database at Children's Hospital of Chongqing Medical University from August 2009 to August 2018 consecutively identified 93 infants who underwent single-stage repair for CoA and intracardiac defects. Inclusive criteria were as follows: diagnosis of CoA and intracardiac defects as confirmed by echocardiogram and enhanced computer tomography (CT) scan, surgical records showing single-stage repair for CoA and intracardiac defects by median sternotomy, and patients undergoing operations were <1 year of age. Exclusive criteria were as follows: patients presenting with isolated CoA, cardiopulmonary bypass (CPB) not performed in single-stage repair for CoA and associated cardiac defects such as patent ductus arteriosus (PDA), patients undergoing operations were older than 1 year old, and reoperation performed due to recoarctation after primary repair.

We collected patient demographics including gender, age, pre-operative transcoarctation pressure gradient, and types of CoA with intracardiac defects. Perioperative conditions such as surgical approaches, operative time, aortic clamping time, and early mortality were collected as well. Early mortality was defined as death directly related to surgery before discharge. The post-operative transcoarctation pressure gradient was collected before discharge. Recoarctation was considered if the transcoarctation systolic pressure gradient was >20 mmHg.

### Surgical Technique

**Figure 1:**
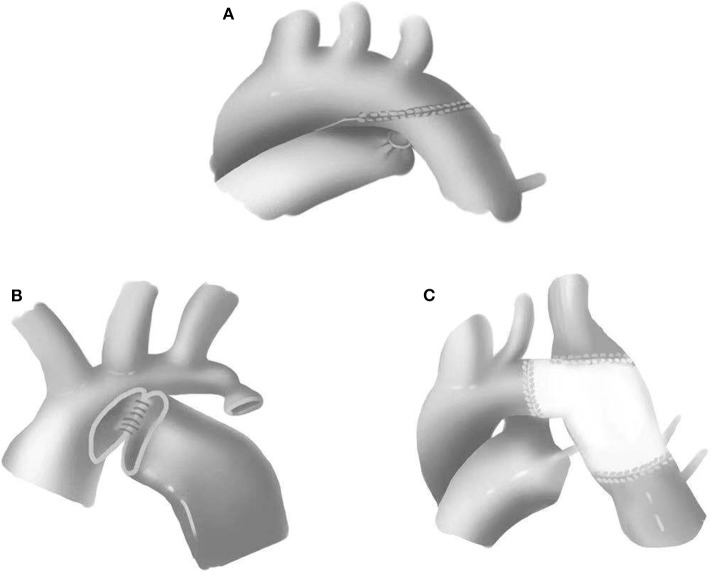
Surgical approaches for coarctation of the aorta. **(A)** Extended end-to-end anastomosis. **(B)** Extended end-to-side anastomosis. **(C)** Patch augmentation.

All cases in this group underwent single-stage repair through median sternotomies. CPB strategy included either deep hypothermic circulatory arrest (DHCA) or selective brain perfusion. To reduce the anastomotic tension and prevent potential recoarctation, the aortic arch, innominate artery, left common carotid artery, left subclavian artery, and descending aorta were completely released. End-to-end anastomosis, extended end-to-end anastomosis, and extended end-to-side anastomosis were performed with the use of 6-0 or 7-0 Prolene suture (Figure 1). Pulmonary artery patch, pericardial patch, or Gore-Tex patch was used for widening the anastomosis when the hypoplasia of the aortic arch was identified. Subsequent repair of intracardiac defects was also performed. The chest would be closed in delay due to severe myocardial edema or hemodynamic instability.

### Post-operative Management and Follow-up

Ventilation and inotropics were monitored after surgery. All of the infants underwent echocardiogram 1 day after surgery and before discharge. Outpatient follow-up was required for all postsurgical survivors. Echocardiogram and enhanced CT scan were ordered during follow-up. Recoarctation was considered in patients showing transcoarctation systolic pressure gradients higher than 20 mmHg. Reinterventions should be considered in patients with significant recoarctation and developmental retardation.

### Statistical Methods

Data were presented as mean ± standard deviation (SD). Statistical significance between pressure gradients at different time points was determined by the Wilcoxon signed-rank test. *P* < 0.05 was considered to be statistically significant. The Kaplan–Meier curve was adopted to calculate the recoarctation-free survival rate. All statistical analyses were performed using the SPSS 18.0 software.

## Results

A total of 93 infants including 61 males and 32 females were identified. The mean age was 93.7 ± 77.2 days (range 1–346 days). Eleven neonates were included in this group, which accounted for 11.8% among all infants, with an average weight of 4.6 ± 1.3 kg (range 2.3–7.6 kg). Associated intracardiac defects included 79 cases of VSD, 64 cases of atrial septal defect (ASD), two cases of double outlet of right ventricular (DORV), two cases of double chamber right ventricles, one case of total anomalous pulmonary venous connection (TAPVC), one case of cor triatriatum, one case of mitral valve prolapse, one case of bicuspid aortic valve, one case of mitral valve dysplasia, and one case of tricuspid valve dysplasia. No Shone's complex was identified in our study.

**Figure 2:**
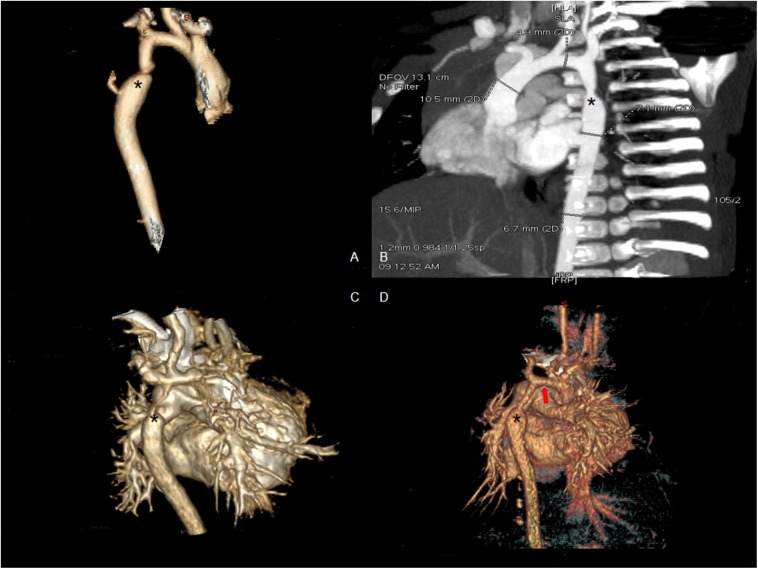
Enhanced CT scan showing coarctation of the aorta before surgery. **(A,B)** Eight-month male with coarctation of the aorta (asterisk shows the coarctation). **(C)** Four-day-old male with severe coarctation of the aorta (asterisk shows the coarctation). **(D)** One-month-old male with coarctation of the aorta and hypoplasia of the aortic arch (asterisk shows the coarctation and red arrow shows hypoplasia of the aortic arch).

**Figure 3:**
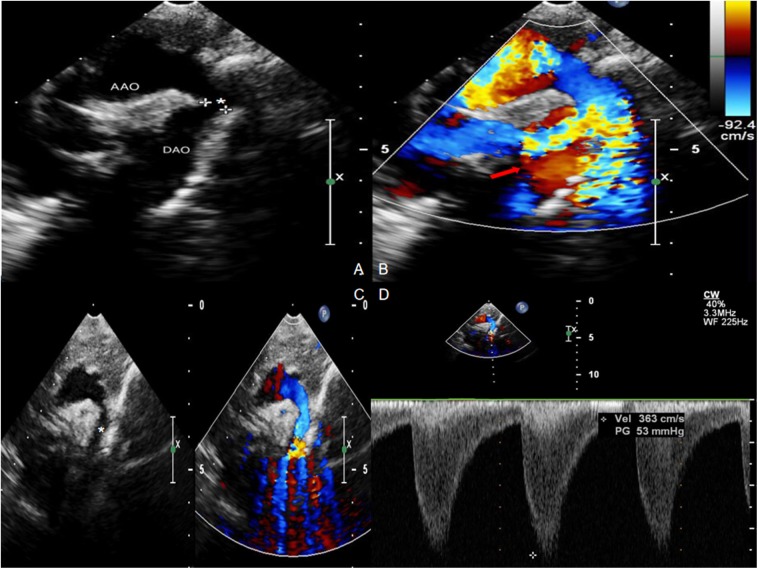
Echocardiogram with Color Doppler Flow Imaging showing coarctation of the aorta and increased blood flow velocity. **(A,C)** Coarctation of the aorta (asterisk). **(B,D)** Increased blood flow velocity (red arrow) with multicolored flow signal.

All infants underwent enhanced cardiac CT (Figure 2) and echocardiogram (Figure 3). Hypoplasia of the aortic arch (CoA with tubular hypoplasia) was found in 23 cases. Coarctation of aortic isthmus (between the left subclavian artery and PDA) was found in 32 cases. Average diameters of the ascending aorta and the narrowest segment, which were measured by reconstruction CT, were 8.90 ± 2.31 and 3.21 ± 1.05 mm, respectively. The mean systolic pressure gradient, which was measured by echocardiogram, was 48.3 ± 17.5 mmHg. The invasive blood pressure gradient between the upper and lower limbs, which was measured after sedation, was 39.6 ± 14.3 mmHg. Missed diagnoses were found in two cases initially diagnosed with VSD and ASD by echocardiogram before surgery, but identified as CoA during surgery. Misdiagnoses of interrupted aortic arch (IAA) were identified in five cases through echocardiogram, but cardiac CT and 3D reconstruction established the correct diagnosis of CoA.

Pre-operative CT scan showed that 63 infants were associated with pneumonia and airway reconstruction suggested airway compression in 22 infants due to the enlarged left atrium and left ventricle. Aortic arch release and end-to-side anastomosis were performed when airway compression was significant. Aortic suspension was also conducted as necessary. Airway compression in these infants was alleviated eventually, and no tracheal stenosis was observed after surgery.

All surgeries were performed through median sternotomies. CPB strategy included either DHCA in 10 cases or selective brain perfusion in others. End-to-end anastomosis was performed in six cases, extended end-to-end anastomosis was performed in 11 cases, extended end-to-side anastomosis was performed in 62 cases, pulmonary patch augmentation was performed in 12 cases, Gore-Tex patch augmentation was performed in one case, and pericardial patch augmentation was performed in one case. Thirteen cases underwent delayed chest closure. The mean operative time and aortic clamping time were 279.0 ± 56.4 and 74.7 ± 25.2 min, respectively.

Complications were found in 20 cases, which accounted for 21.5% in our group. Low cardiac output syndrome (LCOS) was found in nine infants, which was alleviated with the use of peritoneal dialysis and inotropics. Chylothorax was found in one patient and was relieved after chest drainage and parenteral nutrition. Massive pericardial bleeding was found in four cases and was resolved with plasma transfusion and hemostatics. Pneumonia was found in five infants and was also relieved using antibiotics. Wound infection was found in one patient.

**Figure 4:**
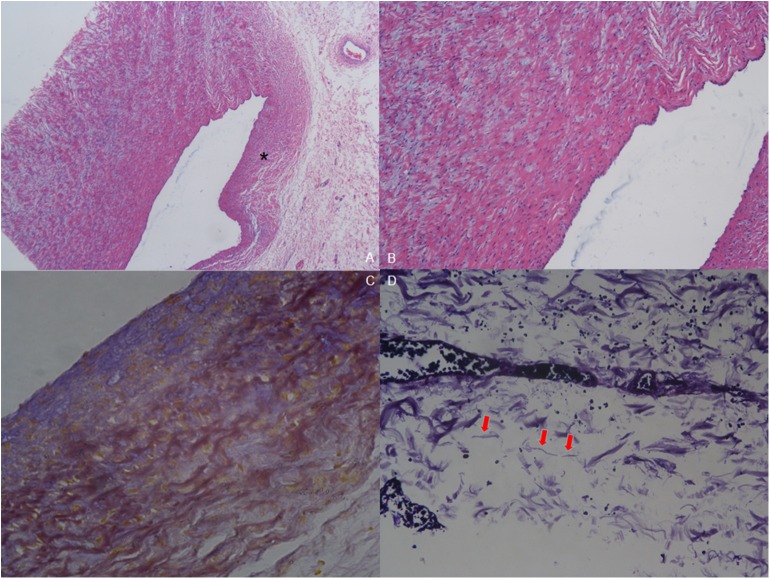
Pathology of resected tissue. **(A)** Hematoxylin-eosin (HE) staining (40×) shows reduction in elastic layers on the PDA-ipsilateral side (asterisk) compared with the PDA-contralateral aortic tissue. **(B)** HE staining of the endothelium of the aortic arch (100×). Intima was absent of normal squamous epithelial cells and wave-like connective fibers. Mucoid degeneration can be observed among the external muscular fibers. **(C)** Elastic fiber staining of coarctation (200×). The muscular fibers are stained yellow to show the wave-like change, and the elastic fibers are stained blue. **(D)** Elastic fiber staining (800×) shows that elastic fibers are broken and not continuous (red arrow).

The pathology of resected tissue showed intermittent violet elastic fibers in the loose tissue of the aortic arch. Meanwhile, hematoxylin-eosin (HE) staining of the aortic arch showed reduction in elastic layers on the PDA-ipsilateral side, as compared with the PDA-contralateral aortic tissue. Intima also lacked normal squamous epithelial cells and wave-like connective tissue. Mucoid degeneration could be observed in the external muscular fibers (Figure 4).

**Figure 5:**
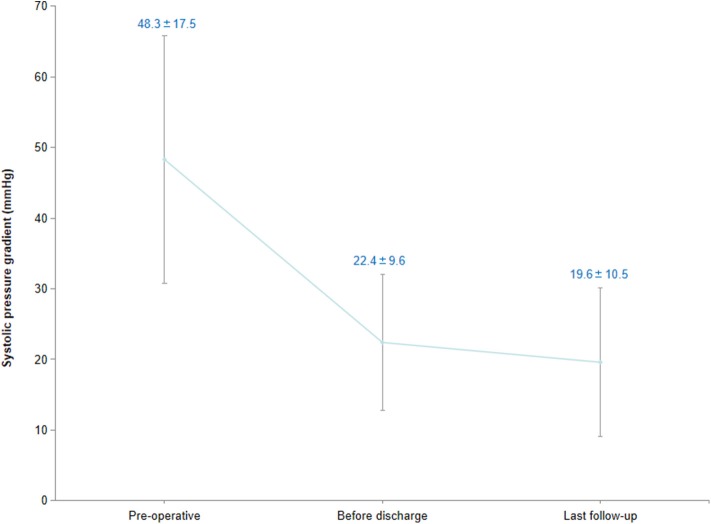
Transcoarctation systolic pressure gradient.

Five infants died after surgery, and the early mortality was 5.4%. Four of them died of post-operative LCOS, and one died of pulmonary infection and respiratory failure. The residual transcoarctation systolic pressure gradient, which was measured by echocardiogram before discharge, was 22.4 ± 9.6 mmHg. Compared with the data prior to surgery, the systolic pressure gradient descended significantly (Figure 5) after surgery (*Z* = 6.72, *P* < 0.01). Minor residual shunt was found in three cases with VSD, but further intervention was not required.

**Figure 6:**
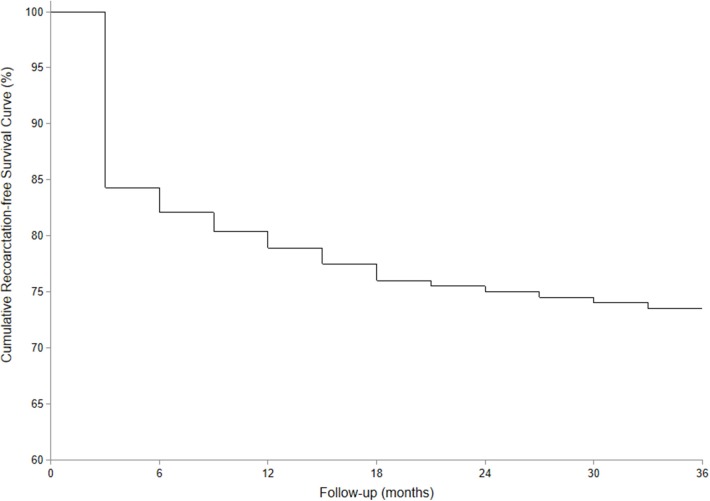
Cumulative recoarctation-free survival curve in the follow-up.

The average follow-up time was 40.0 ± 26.4 months (range, 7–106 months), and there was no late mortality in the follow-up. Both echocardiogram and enhanced CT scan were ordered to evaluate the intracardiac conditions and morphology of the aortic arch. The average transcoarctation systolic pressure gradient in the last follow-up was 19.6 ± 10.5 mmHg, and no significant difference was found compared with the pressure gradient before discharge (*Z* = 1.18, *P* > 0.05). The pressure gradient of 27 infants in the last follow-up was higher than 20 mmHg. No further treatment was necessary in 21 cases with an average pressure gradient of 26.0 ± 4.7 mmHg since there were no significant symptoms and the echocardiogram did not indicate significant recoarctation or left heart dysfunction. The pressure gradient of the remaining six infants, which included two cases of hypoplasia of the aortic arch, was higher than 40 mmHg. In these six infants, four of them suffered significant lower limb retardation and were recommended for balloon angioplasty consultation. The remaining two cases had no obvious symptoms, and their parents were not willing to get reintervention. The recoarctation-free survival rate in the 3-year follow-up was 73.5% (Figure 6).

## Discussion

The currently main surgical strategies (7–9) for CoA associated with intracardiac anomalies in infants include the following: (1) surgical repair of CoA and pulmonary banding (if necessary) at the first stage, followed by subsequent repair of intracardiac defects; (2) single-stage repair of CoA and intracardiac defects with the use of selective brain perfusion or DHCA; and (3) single-stage repair with double incisions including lateral thoracotomy for CoA and median sternotomy for intracardiac defects. In the 1990s, several studies reported primary repair for CoA and second-stage repair for VSD (10, 11). Since non-restricted VSD such as subpulmonary VSD is not self-limited, secondary surgery is often inevitable. Brouwer et al. (11) reported single-stage repair with double incisions for CoA associated with intracardiac defects. Compared with median sternotomy, CPB and hospitalization time were shorter with the use of double incision strategy. However, patients had to be repositioned during the surgery, and hypoplasia of the aortic arch could not be repaired through lateral thoracotomy. To ensure satisfactory exposure of the surgical field, all 93 infants in our group underwent median sternotomy, which was a better alternative especially for patients with hypoplasia of the aortic arch and intracardiac defects.

DHCA provides a satisfactory surgical field exposure and is mainly adopted for patch augmentations in our center. DHCA was conducted in seven cases and no neural complications were found after surgery in this group. Due to improvement of surgical skills, DHCA techniques and post-operative management, the safety of single-stage repair through median sternotomy, which has fewer neural complications in recent years, is established.

The main surgical approaches for CoA consist of end-to-end anastomosis, extended end-to-end anastomosis, extended end-to-side anastomosis, and patch augmentation (12). We mainly performed extended end-to-side anastomosis and pulmonary artery patch augmentation, which accounted for 80% of all cases. Extended end-to-side anastomosis was mainly adopted for repairing the hypoplasia of the aortic arch (13) and widening the anastomosis. Furthermore, with this approach, post-operative recoarctation can be reduced since the aortic arch is reconstructed with autologous arteries, which accommodates for growth in children. The pulmonary artery patch possesses autologous endothelium and has arterial elasticity. Consequently, pseudoaneurysm and thrombus can be avoided with the use of the pulmonary artery patch (14). Using a Gore-Tex patch is simple and time-saving, but it has no potential for growth. Besides, complications such as hemorrhage, recoarctation, and pseudoaneurysm formation after surgery can be often found (15). Therefore, we performed only one Gore-Tex patch augmentation in the early period. In recent years, pulmonary artery patch augmentation has been used more frequently and the outcomes are satisfactory. No post-operative bleeding and pseudoaneurysm formation have been observed.

Transcoarctation pressure gradients higher than 20 mmHg were found in about one-third of hospital survivors in the follow-up. However, most of them had no clinical symptoms and echocardiogram indicated normal left heart function without significant recoarctation. Four of them, whose gradient was higher than 40 mmHg, suffered lower limb retardation and were recommended for balloon angioplasty consultation.

There were two patients with missed diagnosis and five patients of misdiagnosis by echocardiogram. In one case of missed diagnosis, the echocardiogram indicated that the blood flow velocity of the descending aorta was increased, but cardiac CT was not conducted. CoA was finally diagnosed during surgery. The misdiagnosis of IAA in five cases was corrected using cardiac CT. This condition may be related to the narrow and strip-like shape of the coarctation, which is difficult to clarify with echocardiogram. Thus, currently in our center, if echocardiogram suggests CoA, IAA, or increased blood flow velocity of the descending aorta, cardiac CT scan and reconstruction will be recommended.

## Limitations

There are several limitations of our study. This is a retrospective study with potential recall bias, so a randomized controlled trial (RCT) is required to confirm the safety and efficacy of single-stage repair for CoA and associated cardiac defects. The follow-up data in this study are not sufficient, since this study lacks long-term follow-up (5 years or longer). Hypertension is a common complication after CoA repair; however, in this study, we did not collect the data regarding blood pressure in the follow-up. The echocardiograms in our center have only recorded the diameters and pressure gradient of coarctation; however, the Z score, which is used to evaluate the morphology of the aortic arch, is not measured, and therefore, we currently lack quantification for aortic arch development. In the case of associated procedures, the Aristotle Basic Complexity Score (16) defines the primary procedure as the one with the highest complexity. Therefore, in this group, the basic complexity is scored based on the complexity of TAPVC, DORV, mitral and tricuspid valves, or coarctation repair. The average Complexity Basic Score was 7.6; therefore, the overall complexity of this group is limited.

## Conclusion

In conclusion, to avoid secondary surgery in very young patients, single-stage repair for CoA associated with intracardiac defects in infants shows overall satisfactory outcomes in the short- to mid-term follow-up. Mortality is 5.4% for the current study, complications are observed in 20% of the patients, and 4 out of 88 survivors need extra angioplasties due to recoarctation. However, this result is neither applicable to all types of CoA nor to extracardiac defects.

## Data Availability Statement

The datasets generated for this study are available on request to the corresponding author.

## Ethics Statement

Ethical approval of this study was approved by the Institutional Review Board of Children's Hospital of Chongqing Medical University (2019.258). All infants' parents have signed the informed consent after admission.

## Author Contributions

YW: design of the work, acquisition, analysis, interpretation of data, and drafts the manuscript. JL: acquisition, analysis, interpretation of data, and translation of manuscript. CW: design of the work. JZ: helped to perform the elastic fibers staining, analysis, and interpretation of pathology data. LH and CF: acquisition, analysis, and interpretation of CTA data. YY: drafts and translation the manuscript. XJ: design of the work, drafts the work and substantively revises it, and approves the final manuscript.

### Conflict of Interest

The authors declare that the research was conducted in the absence of any commercial or financial relationships that could be construed as a potential conflict of interest.

## References

[1] Bruse JL Khushnood A McLeod K Biglino G Sermesant M Pennec X. How successful is successful? Aortic arch shape after successful aortic coarctation repair correlates with left ventricular function. J Thorac Cardiovasc Surg. (2016) 153:418–27. 10.1016/j.jtcvs.2016.09.018 27776913

[2] Gendera K Ewert P Tanase D Georgiev S Genz T Bambul Heck P. Balloon-expandable stents for recoarctation of the aorta in small children. Two centre experience. Int J Cardiol. (2018) 263:34–9. 10.1016/j.ijcard.2018.02.054 29754919

[3] Martins JD Zachariah J Selamet Tierney ES Truong U Morris SA Kutty S. Rationale and design of long-term outcomes and vascular evaluation after successful coarctation of the aorta treatment study. Ann Pediatr Cardiol. (2018) 11:282–96. 10.4103/apc.APC_64_18 30271019 PMC6146860

[4] Mishima A Nomura N Ukai T Asano M. Aortic coarctation repair in neonates with intracardiac defects: the importance of preservation of the lesser curvature of the aortic arch. J Card Surg. (2014) 29:692–7. 10.1111/jocs.12407 25041795

[5] Plunkett MD Harvey BA Kochilas LK Jeremiah SM James DSL. Management of an associated ventricular septal defect at the time of coarctation repair. Ann Thorac Surg. (2014) 98:1412–8. 10.1016/j.athoracsur.2014.05.076 25149056 PMC5701891

[6] St Louis JD Harvey BA Menk JS O'Brien JE Jr. Kochilas LK. Mortality and operative management for patients undergoing repair of coarctation of the aorta: a retrospective review of the pediatric cardiac care consortium. World J Pediatr Congenit Heart Surg. (2015) 6:431–7. 10.1177/2150135115590458 26180161

[7] Gray WH Wells WJ Starnes VA Kumar SR. Arch augmentation via median sternotomy for coarctation of aorta with proximal arch hypoplasia. Ann Thorac Surg. (2018) 106:1214–9. 10.1016/j.athoracsur.2018.04.025 29753817

[8] Atalay A Pac A Avci T Zengin NI Amac ND Eris D. Histopathological evaluation of aortic coarctation after conventional balloon angioplasty in neonates. Cardiol Young. (2018) 28:683–7. 10.1017/S1047951117002967 29345605

[9] Koletsis E Ekonomidis S Panagopoulos N Tsaousis G Crockett J Panagiotou M. Two stage hybrid approach for complex aortic coarctation repair. J Cardiothorac Surg. (2019) 4:1–5. 10.1186/1749-8090-4-10 19239693 PMC2652448

[10] Kanter KR Mahle WT Kogon BE Kirshbom PM. What is the optimal management of infants with coarctation and ventricular septal defect? Ann Thorac Surg. (2017) 84:612–8. 10.1016/j.athoracsur.2007.03.021 17643644

[11] Brouwer RM Cromme-Dijkhuis AH Erasmus ME Contant C Bogers AJ Elzenga NJ. Decision making for the surgical management of aortic coarctation associated with ventricular septal defect. J Thorac Cardiovasc Surg. (1996) 111:168–75. 10.1016/S0022-5223(96)70413-0 8551762

[12] Lescan M Hornung A Hofbeck M Schlensak C. Custom-made stent grafts for the treatment of pseudoaneurysms after childhood coarctation surgery. Thorac Cardiovasc Surg. (2019) 67:50–4. 10.1055/s-0038-1642629 29772586

[13] Tulzer A Mair R Kreuzer M Tulzer G. Outcome of aortic arch reconstruction in infants with coarctation: importance of operative approach. J Thorac Cardiovasc Surg. (2016) 152: 1506–13.e1501. 10.1016/j.jtcvs.2016.08.029 27692955

[14] Ma ZL Yan J Li SJ Hua ZD Yan FX Wang X. Coarctation of the aorta with aortic arch hypoplasia: midterm outcomes of aortic arch reconstruction with autologous pulmonary artery patch. Chin med J. (2017) 130:2802–7. 10.4103/0366-6999.215279 28936993 PMC5717858

[15] Bertolini A Dalmonte P Toma P Bava GL Corazza G Marasini M. Goretex patch aortoplasty for coarctation in children: nuclear magnetic resonance assessment at 7 years. J Cardiovasc Surg. (1992) 33:223–8. 1572882

[16] Jacobs DJ Daebritz S Comas J Comas J Daebritz S Daenen W. The aristotle score: a complexity-adjusted method to evaluate surgical results. Eur J Cardiothorac Surg. (2004) 25:911–24. 10.1016/j.ejcts.2004.03.027 15144988

